# Hundreds of putatively functional small open reading frames in *Drosophila*

**DOI:** 10.1186/gb-2011-12-11-r118

**Published:** 2011-11-25

**Authors:** Emmanuel Ladoukakis, Vini Pereira, Emile G Magny, Adam Eyre-Walker, Juan Pablo Couso

**Affiliations:** 1 Department of Biology, University of Crete, PO Box 2208, 71409 Heraklio, Greece; 2 School of Life Sciences, University of Sussex, Falmer, Brighton, BN1 9QG, UK

## Abstract

**Background:**

The relationship between DNA sequence and encoded information is still an unsolved puzzle. The number of protein-coding genes in higher eukaryotes identified by genome projects is lower than was expected, while a considerable amount of putatively non-coding transcription has been detected. Functional small open reading frames (smORFs) are known to exist in several organisms. However, coding sequence detection methods are biased against detecting such very short open reading frames. Thus, a substantial number of non-canonical coding regions encoding short peptides might await characterization.

**Results:**

Using bio-informatics methods, we have searched for smORFs of less than 100 amino acids in the putatively non-coding euchromatic DNA of *Drosophila melanogaster*, and initially identified nearly 600,000 of them. We have studied the pattern of conservation of these smORFs as coding entities between *D. melanogaster *and *Drosophila pseudoobscura*, their presence in syntenic and in transcribed regions of the genome, and their ratio of conservative versus non-conservative nucleotide changes. For negative controls, we compared the results with those obtained using random short sequences, while a positive control was provided by smORFs validated by proteomics data.

**Conclusions:**

The combination of these analyses led us to postulate the existence of at least 401 functional smORFs in *Drosophila*, with the possibility that as many as 4,561 such functional smORFs may exist.

## Background

Genome sequencing projects have dramatically expanded our knowledge of genome structure and organization. They have also offered a challenge for identifying functional (that is, translated and biologically relevant) coding sequences in genomes. However, 15 years after the first eukaryotic genome was sequenced [1] and after the complete sequencing of more than a hundred eukaryotic genomes, gene prediction remains a non-trivial exercise [2]. For example, we still do not have the complete set of translated ORFs for all loci in the genome of any higher eukaryote [3,4]. Gene prediction programs use two main computational methods: in the first, *de novo *gene prediction is made using mathematical models that determine the probabilities for all possible intron-exon annotations in a given sequence [3]. The second is based on a comparison between the genome and a known database of cDNA sequences or genes from related organisms. There are several computer programs that identify putative coding sequences (for example, the Critica Suite). These programs are reliable for predicting sequences above a certain length, but are less reliable for predicting functional small open reading frames (smORFs) [5]. smORFs of less than 100 amino acids corresponding to functional genes can escape detection because they may be buried in a pile of 'junk' ORFs formed by chance [5,6]. Yet, smORFs might encode translated and biologically active peptides. It is well known, for example, that short peptides can have important functions, as exemplified by hormones and neuropeptides. As of December 2009, UniProt listed 218,504 entries for peptides of less than 100 amino acids in all organisms, of which 256 are in *D. melanogaster*. These peptides have important functions, such as mating pheromones, peptides involved in energy metabolism, proteolipids, chaperonins, stress protein transporters, transcriptional regulators, nucleases, ribosomal proteins, thioredoxins, metal ion chelators, hormones, antibacterial peptides, short transporter peptides and kinase regulatory subunits. The translation of these peptides has been established by functional and biochemical essays in an *ad hoc *manner over the years. Typically, the peptides that have been identified arise from the processing of a longer protein precursor. This has led to the suggestion that a 'cryptome' might exist, composed of biologically active peptides with their own separate functions resulting from the processing of well-characterized proteins [7]. A more recent development is represented by studies into uORFs, short ORFs found upstream of long ORFs in canonical transcripts. It is known that uORFs can regulate the translation of the downstream main ORF [8]. Studies of these putatively regulatory ORFs in humans, animals, plants and fungi have shown, however, high levels of conservation in the putative amino acid sequence and a ratio of non-synonymous (Ka) to synonymous (Ks) nucleotide substitutions that is less than one [8], while some of these uORFs also show an absence of regulatory effects on the downstream ORF [9,10]. Altogether, these observations suggest that rather than being a mere regulatory region, uORFs can encode functional peptides. A further case that is emerging is that of functional smORFs that encode short peptides and are not included in a transcript containing a long, canonical ORF but have their own, specific, smORF-encoding-only transcript.

A systematic search for smORFs has been undertaken in *Arabidopsis*, and it has been suggested that 3,241 potential translated smORFs exist on the basis of their Ka/Ks index [11]. However, the question of which smORFs belong to functional genes has only been comprehensively addressed in yeast. At first, all ORFs of less than 100 codons (including the initial ATG codon) were excluded from the initial yeast genome annotation [12]. This decision was later justified by the observation that ORFs of 100 to 150 codons include numerous artifactual ORFs [13,14]. In subsequent years many studies examined whether (and how many) smORFs are actually functional. Many yeast smORFs were identified by virtue of their expression, using serial analysis of gene expression (SAGE) [15,16], Northern blotting [17], RT-PCR [18] and ORF tagging. More recently, Kastenmayer *et al*. [19], pooling all published information, concluded that there are 299 functional smORFs in *Saccharomyces cerevisiae*, which is about 5% of the previously annotated protein-coding sequences in this yeast.

The first annotations of the *Drosophila *genome did not set a lower ORF size for coding sequence finding, but it was reported that smORFs are missed because of difficulties in annotation [20]. Such releases eliminated smORFs that did not have additional supporting evidence (for example, presence of protein domains, homology to known proteins). More recent annotations (FlyBase release 5 [21]; as accessed in May 2010) have introduced many putative ORFs between 50 and 100 amino acids but most of these represent isoforms (or truncated cDNAs) of canonical, long proteins (V Pereira and JP Couso, unpublished observations). However, the non-canonical, polycistronic *Drosophila *gene *tarsal-less *(*tal*), encoding smORFs of only 11 amino acids in length has been isolated by standard genetic methods, and its function proven to be mediated by the translated peptides [22-24] even though it had been deemed non-coding initially by the *Drosophila *genome project and previous studies [25]. This single yet non-biased functional example showed that, in principle, very short but functional smORFs may have escaped from genome annotation programs but still be present in the genome of *Drosophila*. Furthermore, a *tal *gene family has been identified in arthropods, containing homologous smORFs of between 10 and 13 codons that are translated into peptides that function during development [22,26]. These results show that very short smORFs whose functionality is completely independent of a long ORF do exist in higher eukaryotes.

**Figure 1:**
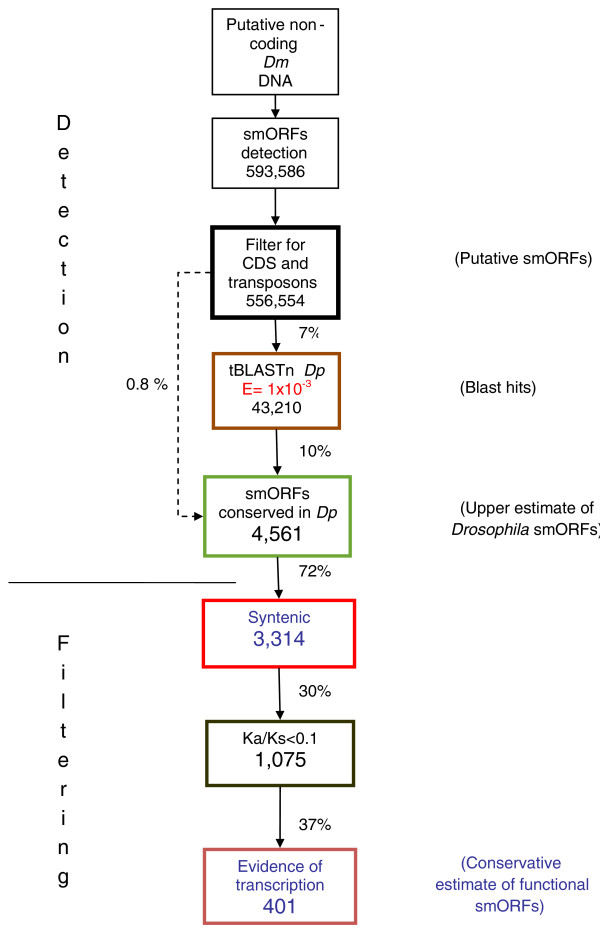
**Search pipeline for *Drosophila *smORFs**. Diagram of the smORF search pipeline followed in this study. The percentages of smORFs passing each filter are indicated. For full details, see Results and Materials and methods. CDS, coding DNA sequence; Dm, *Drosophila melanogaster*; Dp, *Drosophila pseudoobscura*; Ka/Ks, ratio of non-synonymous (Ka) to synonymous (Ks) nucleotide substitution.

Here we report the results from a systematic search for putatively functional smORFs in the *Drosophila *genome. Our search pipeline is presented in Figure 1. In brief, we scanned putative non-coding regions of the *Drosophila *genome for ORFs of less than 100 amino acids conserved between *D. melanogaster *and *Drosophila pseudoobscura*, two closely related species separated from their common ancestor by 25 to 55 million years [27]. We investigated whether these putative smORFs were transcribed and had a ratio of non-synonymous to synonymous substitution indicative of protein sequence conservation. Our analyses suggest that there are at least 401 functional smORFs in *Drosophila*, which would represent almost an additional 3% to the 13,907 protein-coding genes that have been annotated so far (FlyBase release 5 [21]; as accessed in October 2011). This fraction is in line with the estimates for uncharacterized smORFs in yeast and mouse (5% of canonical genes) [5,28] and our adjustment of a previous estimate for *Arabidopsis thaliana *[11]. Finally, we provide a validation of our bioinformatics search by analyzing examples of previously annotated smORFs for which evidence of translation exists. This validation reinforces the precaution that 401 may actually be an underestimate; the upper estimate from our data suggests that up to 4,561 functional smORFs may exist in *Drosophila*.

## Results

### Initial search for smORFs

We searched the non-exonic DNA of the euchromatin portion of the *D. melanogaster *genome for smORFs using two criteria. First, the length of the ORF had to be between 30 and 300 bp. Second, a start and a stop codon had to be in frame. We therefore restricted our search to single exon smORFs. If a sequence met these criteria, we counted it as a putative smORF. We restricted our analysis to intronless smORFs because fragmentation of exons with introns would further reduce the size of the coding sequence and would greatly increase the difficulty of detecting smORFs. Underestimation of the number of smORFs due to this exclusion is not expected to be high because there is a positive correlation between the length of a protein and the number of introns [29]; smaller coding sequences tend to have fewer introns. We observed 593,586 putative smORFs in the euchromatin of the *D. melanogaster *genome. The putative smORFs were distributed across chromosomal arms roughly in proportion to the length of each arm's euchromatin (FlyBase release 5 [21]; as accessed in December 2009). Chromosomal arm 3R, which is the largest, has the highest number of smORFs (24%), and chromosomal arm 4, which is the smallest, has the lowest number of smORFs (6.6%) (Table 1). Assuming a random even distribution, this produces an estimate of one putative smORF every 400 bp of euchromatic DNA (this was estimated by dividing the length of the *D. melanogaster *genome, considering both strands, by the number of putative smORFs: 2 × 120,388 kb/593,586.

**Table 1:** smORF numbers

Chromosomal arm	Euchromatin (bp)	Total number of smORFs	Number of smORFs after filtering for CDSs and transposons	*Dm *smORFs conserved in *Dp*
1 (X)	22,422,827	114,896	107,288	584
2L	23,011,544	118,333	111,404	1,010
2R	21,146,708	94,762	87,249	653
3L	24,543,557	119,069	112,256	878
3R	27,905,053	142,593	135,420	1,431
4	1,351,857	3,933	2,937	5
Total	120,377,990	593,586	556,554	4,561

Distribution of putative smORFs across the chromosomal arms of *D. melanogaster *(*Dm*), showing the number of smORFs after filtering for coding DNA sequences (CDSs) and transposons, and the number of *Dm *smORFs conserved in *D. pseudoobscura *(*Dp*). The euchromatin content of each chromosomal arm is indicated in nucleotide base pairs.

We do not expect all of these smORFs to be functional, as short ORFs can easily appear by chance. In fact it has been suggested that there might be selection for stop codons in non-coding DNA to reduce the length of aberrantly translated DNA sequences, thus producing a bias towards short ORFs in non-functional but transcribed DNA [30]. We removed any putative smORF that showed significant similarity within databases of *D. melanogaster *transposons and annotated coding sequences, using BLASTn. This analysis reduced the total number of putative smORFs to 556,554 (Figure 1, Table 1).

As a control for this smORF population we extracted from the same non-exonic *D. melanogaster *DNA a pool of 'reverse' smORFs running from stop to start (see Materials and methods for details). This extraction produced some 800,000 'reverse' smORFs and from them we randomly selected a sample of some 54,000 with the same size distribution as the 556 K putative smORF population. This control sample is thus composed of random DNA sequences comparable, and non-overlapping, to our putative smORF population. Submitting this control sample to the same filters as our putative smORFs allows us to ascertain if the smORFs selected by our bioinformatics analyses differ from what could be expected by random chance.

To investigate the presence of these putative smORFs outside *D. melanogaster*, we performed a BLAST of each smORF against the *D. pseudoobscura *genome. The two fly species diverged approximately 25 to 55 million years ago [27] and are sufficiently divergent that we do not expect to detect significant similarity between putatively neutral sequences. Any significant similarity is therefore expected to be due to active sequence conservation by natural selection. However, in a protein-coding sequence nucleotide substitutions at synonymous sites do not alter the amino acid sequence and are expected to be largely hidden from selection [31]. In a small ORF, synonymous substitutions create significant noise, covering the signal of conservation at the nucleotide level, and even preventing BLAST from detecting similarity between sequences [32]. At the amino acid level, however, the noise of non-conserved synonymous sites is eliminated. We therefore compared the *D. melanogaster *amino acid sequence of the putative smORFs with all possible translations of the *D. pseudoobscura *genome, using tBLASTn. We employed a false discovery rate (FDR) framework to estimate the proportion of smORFs that would have a given level of sequence similarity to the *D. pseudoobscura *genome purely by chance. To determine the cutoff tBLASTn E-value that we should employ, we performed a BLAST with both our 54,000 sample of random control 'reverse' smORFs and a similar sample of canonical exonic *Drosophila *sequences (with the same size distribution as these controls, and hence as the 500 K putative smORF pool) against the *D. pseudoobscura *genome, and then compared the E-value distributions of both pools. Thus, a cutoff of E = 0.05 (as a standard value for biological significance) produces a FDR of nearly 10% (9.2%); a cutoff of E = 1 × 10^-3 ^produces an FDR of nearly 7% (6.8%), and a cutoff of E = 1 × 10^-5 ^produces and FDR of 5%. Although in any case the FDR remains acceptable, we decided to opt for a cutoff value of E = 1 × 10^-3 ^(7% FDR) as a compromise between specificity and sensitivity, while also taking into account the tendency of BLAST to assign low scores to short sequences (the shorter the region of similarity, the more likely it could have arisen by chance). This tendency is illustrated by a shift in the size distributions of the canonical exonic sequences that pass each of these E-value cutoffs. It can be observed that decreasing the E-value cutoff discriminates against small sequence sizes.

**Figure 2:**
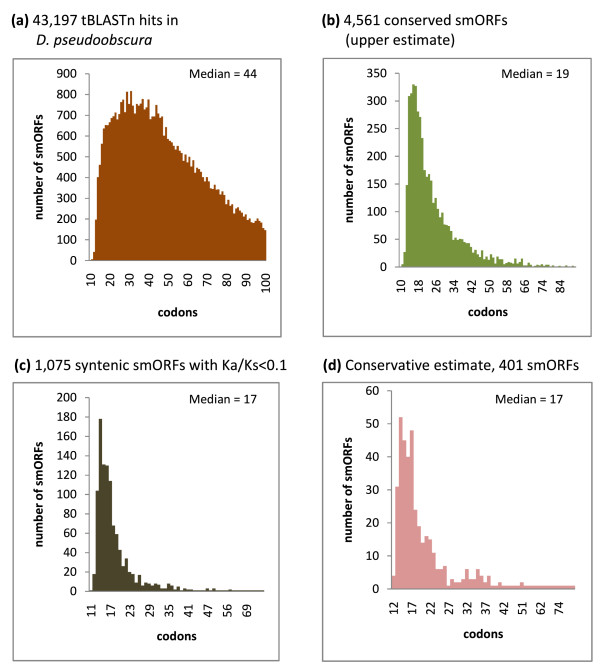
**Size distributions of different pools of smORFs**. The size distribution of different pools of smORFs is represented according to their length in codons. Medians are indicated. **(a) **43,197 smORFs with tBLASTn hits with E-value < 1 × 10^-3 ^representing putative smORFs with some kind of sequence conservation in *D. pseudoobscura*. Mean size = 44 codons, standard deviation = 12. **(b) **4,561 putative smORFs with conservation of sequence and start and stop codons in *D. pseudoobscura*, representing our upper estimate for the number of smORFs in *Drosophila*. Mean size = 25 codons, standard deviation = 12. **(c) **1,075 smORFs with syntenic conservation, and start and stop codons in *D. pseudoobscura*, and with a Ka/Ks (ratio of non-synonymous (Ka) to synonymous (Ks) nucleotide substitution) score < 0.1. Mean size = 19 codons, standard deviation = 8. **(d) **401 smORFs with conservation of sequence, and start and stop codons in *D. pseudoobscura*, with a Ka/Ks score < 0.1, and also present in transcribed regions, representing our conservative estimate. Mean size = 21 codons, standard deviation = 12. For a statistical analysis of the differences between these distributions, see Additional file 1.

**Figure 3:**
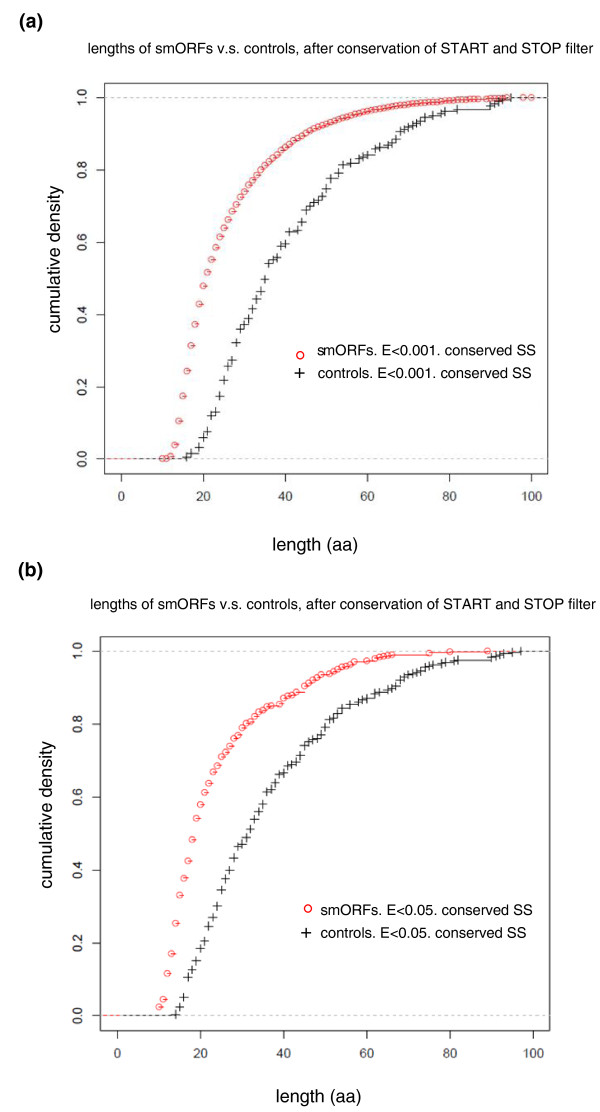
**Cumulative size distributions of smORFs with conserved start and stop codons**. **(a, b) **Size distributions (represented as cumulative graphs) for the putative *D. melanogaster *smORFs with tBLASTn E-value < 1 × 10^-3 ^(a) or < 0.05 (b), and conserved, in-frame start and stop codons in *D. pseudoobscura *(SS), and their respective controls composed of reverse stop-to-start control 'smORFs' passing the same filters. The candidate 'real' smORF distributions are very different from the controls representing random short DNA sequences. For a statistical analysis of the differences between these distributions, see Additional file 1.

Applying this cutoff of E < 1 × 10^-3 ^for the tBLASTn filter dramatically reduced the pool of 556 K putative smORFs to 43,210 (Figure 1). This new pool of 43,210 putative smORFs producing tBLASTn hits in *D. pseudoobscura *has a mean length of 44 codons, with a standard deviation of 22 codons (Figure 2a). However, these may not necessarily represent conserved functional coding sequences, but pseudogenes or other conserved elements in the *Drosophila *genome [33-35]. We therefore designed a further filter to investigate whether the conserved tBLASTn hits in *D. pseudoobscura *formed part of a conserved ORF in the same frame as the putative smORF in *D. melanogaster*. tBLASTn is a local alignment algorithm that aligns only the most similar regions between two sequences rather than the whole sequence. To overcome this, we extracted from the *D. pseudoobscura *sequence a further 300 bp immediately upstream and downstream of the tBLASTn hit (since the hit may correspond to the 5' end of the smORF, or the 3' end, or anything in between). The resulting 600+ nucleotide sequence (5' 300 nucleotides + tBLASTn hit + 300 nucleotides 3') was then re-aligned to the initial *D. melanogaster *smORF using ClustalW, and finally, start and stop codons in frame with the *D. melanogaster *smORF were sought in the re-aligned *D. pseudoobscura *sequence. This led to a dataset of 4,561 sequences that are conserved as smORFs between both species (Table 1, Figure 1). In the genome of *D. pseudoobscura *the remaining 38,250 smORFs either shared a start codon but did not have a stop codon in-frame, or shared a stop but not a start codon, or, finally, simply shared similarity with an array of codons that did not form an obvious ORF. Some of these similar regions might belong to uncharacterized 'orphan' exons not ascribed to a main transcript yet, as found by genome-wide studies of transcription [36,37]. The population of 4,561 conserved smORFs has a mean length of 25 codons, with a standard deviation of 12 codons. Interestingly, this average is shorter in ORF size than the previous pool of smORFs with tBLASTn hits only, and their size distributions appear to be different as well (Figure 2a, b). Both a Mann-Whitney U test and a Kolmogorov-Smirnov goodness-of-fitness test indicate that these differences are statistically significant (*P *< 2.2e-16; Additional file 1). To test whether this difference could be obtained by chance, we subjected our pool of control 'reverse' smORFs to the same filters of tBLASTn and start and stop conservation. The resulting pool of filtered control smORFs has a very different size distribution than that of our 4,561 conserved smORFs according to both Mann-Whitney U test and Kolmogorov-Smirnov goodness-of-fitness test (W = 182,856, *P*-value < 1e-15, and D = 0.4546, *P*-value < 1e-15, respectively; Figure 3a; Additional file 1). The standard interpretation of these results is that our 4,561 conserved smORFs belong to a population of sequences that is significantly different from both the pool of sequences with tBLASTn hits only, and a pool of random short DNA sequences that pass the same filters purely by chance.

### Patterns of evolutionary conservation

Although this conservation in itself would seem potent evidence of functionality, there is still the possibility that some of these positives might still be due to chance. To eliminate spurious cases where sequence similarity was not due to true homology (which would undermine both the previous search and subsequent filters) we examined whether the *D. pseudoobscura *hits were located in genomic positions that are syntenic (homologous) with respect to the original *D. melanogaster *smORFs. Such synteny analysis reveals that the great majority (3,314 out of 4,561, or 72%) of the smORFs conserved in *D. pseudoobscura *are actually conserved in syntenic positions. A lack of synteny does not definitely exclude smORFs as non-functional (as smORFs may have been subjected to individual translocation within the genome), but the occurrence of synteny guarantees that similarity is due to homology and enhances the case for conservation being due to functionality.

**Figure 4:**
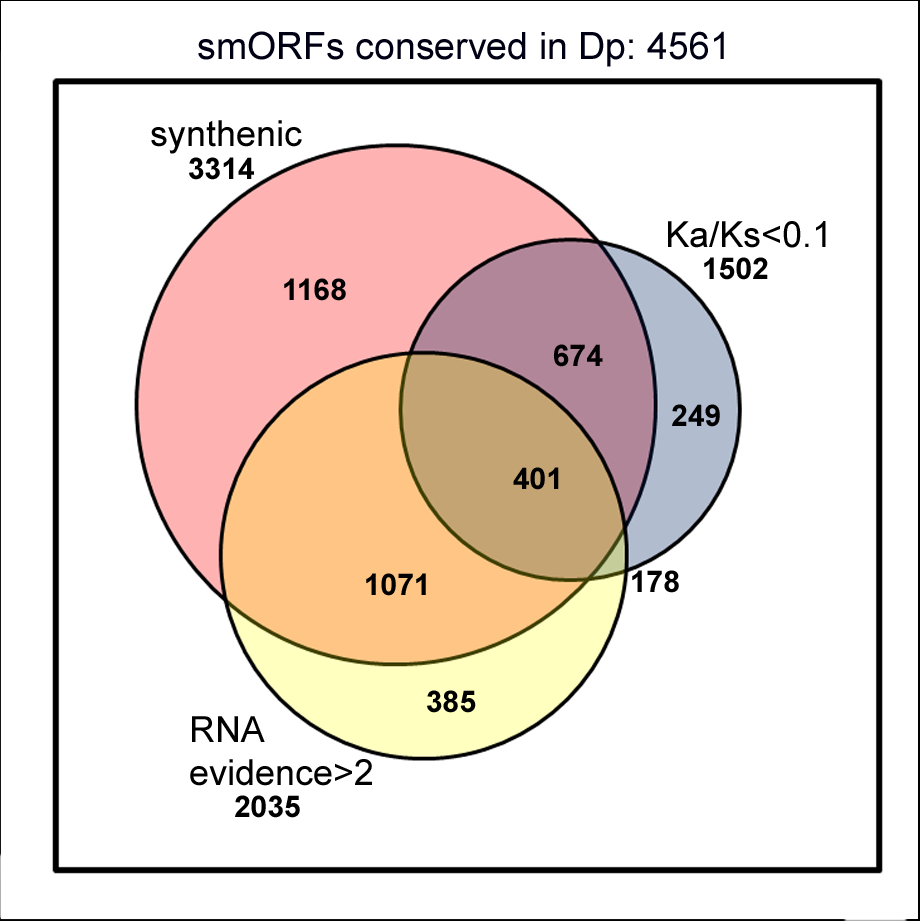
**Distribution of the 4,561 smORFs conserved in *D. pseudoobscura***. Venn diagram representing the distribution of the smORFs with start and stop codons in *D. pseudoobscura *and passing each of the different validation filters, and their combinations. Each circle is proportional to the size of the population it represents. Dp, *D. pseudoobscura*; Ka/Ks, ratio of non-synonymous (Ka) to synonymous (Ks) nucleotide substitution.

Next we assessed what kind of sequence conservation these syntenic smORFs display. A hallmark of evolution in protein coding sequences is a difference in the rate of synonymous (Ks) and non-synonymous (Ka) substitution. The former is expected to be substantially above the latter such that Ka/Ks is expected to be less than one [38]. However, the empirical value of this yardstick for short sequences is debatable, as the original authors observed a high frequency of false negatives amongst coding exons of less than 100 amino acids [38], and reciprocally, it might be argued that such short sequences could also obtain low Ka/Ks scores simply due to chance. We therefore applied a FDR framework (see Materials and methods) and observe that a Ka/Ks value < 0.1 would limit such false positives to 10% in sequences shorter than 100 amino acids. Applying this stringent Ka/Ks limit, we observe that 1,075 of the syntenic smORFs (Figures 1 and 4, Table 2) display a pattern of sequence conservation indicative of protein sequence conservation and significantly different from the conservation shown by random short sequences.

**Table 2:** Ka/Ks analysis of 4,561 smORFs conserved in *D. pseudoobscura*

Ka/Ks	Number of smORFs
0	1,285
< 0.1	1,502
< 1	3,370
> 1	314
NA	877
Total	4,561

Distribution of the 4,561 smORFs conserved in *D. pseudoobscura *according to their ratio of non-synonymous (Ka) to synonymous (Ks) nucleotide substitution. NA, Ka/Ks cannot be computed.

### Transcription of smORFs

As a further test of functionality we investigated whether the conserved smORFs are transcribed using transcription data from *D. melanogaster*. Manak *et al*. [36] investigated the transcriptome of *D. melanogaster *during embryonic development by hybridizing RNA extracted from embryos to a tiling microarray of genomic DNA with an average probe size of 200 bp. They found that 7% of the intergenic *Drosophila *DNA, with no annotated genes, is in fact transcribed. They termed these non-annotated transcribed fragments 'transfrags'. More recently, the modENCODE project has released data obtained with deep RNA sequencing (RNASeq) for embryonic stages as well. We constructed a database in which we integrated all genomic coordinates of transcribed fragments from these two sources (see Materials and methods). We then compared the genome coordinates of all 4,561 conserved smORFs with this database. We found that 2,942 syntenic smORFs overlap transcribed fragments in the right 5' to 3' orientation. However, obtaining such experimental evidence of transcription is not immune to experimental artifacts and false positives. To minimize such cases, we applied the conservative criterion of considering only those cases where multiple evidence of transcription existed (see Materials and methods). Following this criterion, 2,035 smORFs appear to be transcribed. Of these, 1,472 are conserved in syntenic positions, and of these, 401 have Ka/Ks < 0.1 (Figures 1 and 4).

These 401 smORFs have therefore a high probability of being functional, defined as being i) transcribed (by virtue of their overlap with transcribed fragments in the right orientation), ii) translated (by virtue of their low Ka/Ks scores), and iii) biologically relevant genomic units (by virtue of their syntenic conservation as a smORF in *D. pseudoobscura*). Thus, 401 constitutes our first estimate for the number of putatively functional smORFs in *Drosophila*. This number represents approximately 2.9% of the total number of currently annotated protein-coding genes in *Drosophila *(13,907).

A further scrutiny of overlaps between smORFs and transcribed regions reveals that only 14 transcribed fragments overlap more than one smORF, suggesting that 28 smORFs are arranged in 14 polycistronic double-smORF-encoding transcripts. Thus, we surmise that our 401 smORFs may belong to 387 different smORF-encoding transcripts (373 single-smORF-encoding plus 14 double-smORF ones). However, this preliminary analysis does not exclude the possibility that some of our smORFs may belong to new exons of previously annotated transcripts encoding long proteins, which would then be revealed as polycistronic. The precise mapping of these 401 smORFs to genes will require detailed manual curation of genome annotation and, most likely, experimental data (see Discussion).

### Attempted extension of the smORF search

**Figure 5:**
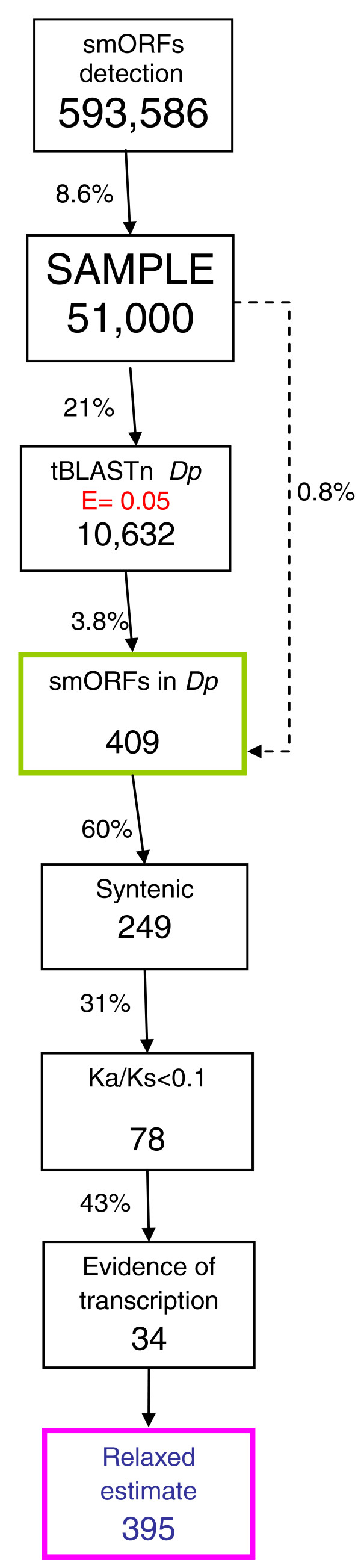
**Relaxed pipeline for smORF search**. Pipeline for search of smORFs with the lowered E-value < 0.05 threshold for the tBLASTn filter. Despite an initial higher percentage of smORFs passing this filter, subsequent results are similar to those obtained by the initial stricter pipeline shown in Figure 1. For details see text and Materials and methods. Dp, *D. pseudoobscura*; Ka/Ks, ratio of non-synonymous (Ka) to synonymous (Ks) nucleotide substitution.

Due to the tendency of tBLASTn to assign low values to short sequences (Additional file 2), and because the ORFs of the *tal *gene obtain E-values as high as 0.87 in tBLASTn (see below), we wanted to investigate whether we might have excluded many functional smORFs by setting the stringency too high in our search for conserved smORFs between *D. melanogaster *and *D. pseudoobscura*. Thus, we tested the effect on our analysis of lowering the E-value threshold in the tBLASTn from 0.001 to 0.05 (which still only produces a 10% FDR according to our tests with canonical exons and control reverse smORFs; see above). Because this 'relaxed' search would generate so many more significant hits, we randomly selected 51,000 smORFs from the 593,586 putative smORFs that we initially found (8.6% of the total; 10,000 from each major autosomal arm and from the X chromosome, and 1,000 from the fourth chromosome; Figure 5). As before, we filtered these smORFs for coding DNA sequences and transposons before searching for conservation in the genome of *D. pseudoobscura *with the new E-value of 0.05. We found 10,632 smORFs that shared similarity at the amino acid level with regions in *D. pseudoobscura *(Figure 5). Of these relaxed 10,632 smORFs, 409 also had conserved in-frame start and stop codons. Of these 409 smORFs with conserved amino acid sequence and ORF structure, nine were difficult to align at the DNA level, or showed a small conserved part and some non-conserved parts. The remaining 400 seem to be good candidates for functional smORFs (that is, 0.8% of the initial 51,000 sample), and just as with the previous stricter analysis their size distribution is significantly different from both the starting pool and what could be obtained by chance (Figure 3b; Additional file 1).

Of these 400, 34 smORFs passed the subsequent filters of syntenic conservation, Ka/Ks < 0.1, and multiple evidence of transcription. Interestingly, the smORF numbers passing each filter are very similar to those obtained in the previous stricter search with an E-value cutoff of 0.001 (Figures 1 and 5): 0.8% of the smORFs detected in *D. melanogaster *were conserved as smORFs in *D. pseudoobscura*, while the estimated number of total functional smORFs in the *Drosophila *genome obtained by extrapolating from the 8.6% 'relaxed' sample is 395, again very close to the actual conservative estimate, 401, obtained in the 'strict' search (Figures 1 and 5).

Altogether, these results indicate that a relaxed search increases neither the conservative pool of candidate smORFs nor the upper estimate for functional smORFs. The subsequent filters in the pipeline, starting with conservation as a smORF in *D. pseudoobscura*, are stringent enough to purify an inflated input.

### Validation of search with examples of translated smORFs

Although different types of analysis and filters seem to converge on a lower estimate of around 400 functional smORFs in *Drosophila*, it would be interesting to independently gauge the accuracy of our pipeline. For this, we collected examples of putative smORFs annotated prior to our analysis (and thus excluded from the pool of non-coding sequences that was the starting point of our search) for which firm evidence of translation for their encoded peptides exists, in the form of proteomic data. These translated and annotated smORFs form a sample of 25 smORFs and the results of submitting them to the filters included in our pipeline are presented in Table 3. Twenty-one translated smORFs pass the filter of tBLASTn with E = 0.001, whereas all 25 have multiple evidence of transcription, and 15 display Ka/Ks < 0.1. Interestingly, only 13 of the peptides are conserved in syntenic positions in *D. pseudoobscura*, and altogether, only 8 of these proteomics-validated smORFs pass all our filters. These results reinforce the notion that our pipeline is accurate but also stringent, and would seem to corroborate our labeling of 401 as conservative and 4,561 as upper estimates for the number of functional smORFs in the *Drosophila *genome.

**Table 3:** Filtering of smORFs with evidence of translation

		Pipeline filters			
		
Gene name [reference]	Length (codons)	tBLASTn E-value (pass if < 10^-3^)	Ka/Ks (pass if < 0.1)	Syntenic to *D. pseudoobscura*	RNASeq overlaps (pass if > 1)
*Akh *[63]	79	2e-22	0.04	**False**	12
CG13551 [64]	87	6e-14	**0.18**	**False**	12
CG14235 [64]	96	5e-42	0.06	True	12
CG17127 [64]	98	4e-42	0.05	True	10
CG30415 [65]	82	4e-36	0.05	**False**	12
CG32039 [64]	82	1e-15	0.06	**False**	12
CG3321 [64,65]	81	6e-23	0.05	True	12
*Cype *[65]	77	8e-26	**0.18**	True	12
*Dbi *[64]	86	7e-39	**0.48**	True	4
*Dro *[66]	64	4e-17	**0.32**	True	9
*Drs *[66]	70	**NA**	**NA**	**Na**	9
*IM1 *[66]	45	1e-12	0.09	True	9
*IM2 *[63,66]	45	9e-14	0.00	True	9
*IM4 *[63,66]	42	**0.075**	**0.11**	**False**	12
l(2)06225 [64,65]	99	3e-45	0.00	True	10
*Mtk *[66]	52	7e-11	**0.27**	True	12
*Nplp2 *[63]	86	**8.4**	0.07	**False**	11
*Nplp3 *[63]	90	1e-10	**0.14**	**False**	7
*Nplp4 *[63]	64	**NA**	**NA**	**NA**	12
*RpL38 *[67]	70	8e-34	0.00	**False**	8
*RpS21 *[67]	83	8e-36	0.02	True	12
*RpS27 *[67]	84	2e-44	0.00	**False**	12
*RpS28b *[64,65]	65	6e-23	0.00	True	12
*smt3 *[64]	90	2e-47	0.00	**False**	12
*sun *[64]	61	1e-16	**0.16**	True	12
Number of smORFs passing each pipeline filter (out of a total of 25)		21	15	13	25

Filtering of previously annotated smORFs with proteomics evidence of translation. References cited in the first column include evidence of translation for each of these smORFs. Entries in bold do not pass the corresponding filter. Ka/Ks, ratio of non-synonymous (Ka) to synonymous (Ks) nucleotide substitution; NA, not available.

## Discussion

In the pre-genomic era the number of genes per genome was predicted based on different techniques, and sometimes extrapolation from other species adjusted by the perceived 'complexity' of a given species. Thus, the human genome was predicted to contain over 100,000 genes [39]. Surprisingly, after genome sequencing and genome annotation this number fell to a quarter of this estimate [40]. In *Drosophila*, the annotation indicated 13,907 protein-coding genes mapped to chromosomes (FlyBase release 5 [21]; as accessed in October 2011), which is in between old estimates based on number of chromosomal bands and saturation mutagenesis of defined regions (5,000 genes) [41], and those based on estimation of non-repetitive DNA content (80,000 genes) [42], as reviewed in [43]. How can we accommodate a large number of functions with a small number of genes? Several answers have been proposed: complex genetic interactions, alternative splicing, and non-protein coding genes [44]. Another less discussed possibility could be that non-canonical coding sequences escape detection from genome annotation pipelines. Already an overwhelming number of RNAs in humans and mice are deemed 'non-coding' (ENCODE data). It could well be that many of these actually encode translated and functional smORFs. For example, the putatively non-coding gene HAR1F [45], which has undergone evolutionary acceleration in humans and is therefore considered as a candidate to participate in the acquisition of human-specific traits, contains several smORFs (JP Couso, unpublished observations). In fact, if putative smORFs were distributed evenly every 400 bp on average, as we find in *Drosophila*, most non-coding RNAs must contain smORFs.

Even though programs for yeast genome annotation initially excluded sequences coding for less than 100 codons [1], yeast remains the best-studied genome for the presence of smORF genes [46-49]. smORF searchers used computational methods for an initial screening of small ORFs and subsequently they tested whether the smORFs are expressed, using a variety of expression identification methods [18]. More recently, Kastenmayer *et al*. [19] collected all potential smORFs from the literature with evidence of either translation or transcription and concluded that the *S. cerevisiae *genome has 299 potentially functional smORFs, which would represent 5% of the genes in yeast. Of these, at least 168 (67% of those tested; Table 4) were found to have evidence of translation into peptides.

**Table 4:** Comparison of smORF searches in different eukaryotic organisms

Organism	smORFs	**Percentage of total**^**a**^	**Experimentally verified**^**b**^
*Saccharomyces*	299	5%	67% (247)
*Arabidopsis*	941	5%	ND
*Mus*	1,240	5%	56% (25)
*Drosophila*	401	3%	ND

Comparison of different smORF searches showing the number of smORFs identified in *S. cerevisiae *[19], *A. thaliana *[11], *M. musculus *[28] and *D. melanogatser *(this study). ^a^The fraction of smORFs relative to previously annotated protein-coding genes. ^b^The proportion of smORFs with direct experimental evidence of translation and/or function. Numbers in parentheses are the actual numbers of smORFs tested experimentally. ND, not determined.

A recent study of the genome of *A. thaliana *revealed 3,241 smORFs that show evidence for either transcription or purifying selection [11]. This estimate is an order of magnitude higher than in yeast; however, Hanada *et al*. [11] reach a number of 3,241 smORF genes by pooling together smORFs with evidence of either transcription or amino acid sequence conservation (a version of the Ka/Ks < 1 test). We would consider this number is therefore a maximum estimate, similar to our 4,561 conserved smORFs. However, Hanada *et al*. point out that the actual number of smORFs with evidence of both transcription and amino acid conservation is 941. This is about 5% of the *Arabidopsis *genes, equal to the fraction postulated in yeast. To date no functional validation of these estimates has been reported.

The study of Frith *et al*. [28] used a modification of the CRITICA gene-detection program suite, originally designed for prokaryote genomes, to detect smORFs in the RIKEN *Mus musculus *cDNA collection. CRITICA's entry point to identify coding sequences is sequence similarity between species, and in particular conservation of coding content of putative ORFs. This study is thus comparable to our own pipeline, in that sequence similarity, coding conservation and evidence of transcription are considered indicators of smORF functionality, and used as filters for their detection. It is interesting, therefore, that the volume of putative new, smORF-encoding sequences found in mouse by Frith *et al*. is again very similar, at 5% of the already annotated fraction of canonical genes with ORFs longer than 100 amino acids. The total number of smORFs (1,240) upon which this fraction is based might be an underestimate, as small cDNAs tend to be eliminated from cDNA collections (as they may represent truncated cDNAs), but Frith *et al*. [28] detect no bias in the size distribution of their smORF-containing cDNAs. In that study, the authors assessed the translation of a sample of these new smORFs, and found evidence of translation for 14 out of 25 smORFs assessed (56%), a fraction very similar to that found in *S. cerevisiae *(67%). It is important to consider that the methods applied (green fluorescent protein tagging in mouse and flies, and a mixture of tagging and proteomics in yeast) can still produce false negatives. Even after applying these fractions as correctors for the total number of real smORFs in eukaryotes, the numbers still hover at around 2 to 3.5% of annotated protein-coding genes, that is, several hundred new coding sequences per organism (Table 4).

The promising results obtained by these three smORF searches previously carried out in other eukaryotes (summarized in Table 4) prompted us to carry out a search for smORFs in *Drosophila*. The *Drosophila *genome is considered as one of the best annotated [20,50-53], yet our study suggests that a remarkably similar fraction of smORFs as in other eukaryotes might have escaped detection by genome annotation efforts and might be an important source of functional genes (Table 4). Programs for *Drosophila*'s genome annotation do not completely omit genes with smORFs from their predictions, but it had been suggested that short coding sequences might escape the cutoff thresholds of the programs [20]. Recently, a lowering of this 100 amino acid threshold has led to the addition of some 700 putative smORFs above 50 amino acids in length (FlyBase release 5 [21]; as accessed in May 2010), but most of these belong to isoforms or truncated cDNAs of canonical long genes, and do not overlap with the smORFs we identify here (V Pereira and JP Couso, unpublished observations). Still, we have corroborated that at least 25 of these seem to correspond to *bona fide *smORFs, with proteomic evidence of translation and sizes ranging from 42 to 99 amino acids (Table 3). Finally, we have recently characterized the *tal *gene, which encodes smORFs of 11 to 32 amino acids, and shown that these peptides carry out important cell signaling functions during development [22-24]. Adding to these examples, we provide here evidence for the existence of hundreds more functional smORFs.

### How many *Drosophila *smORFs are actually functional?

Sequence conservation is an accepted hallmark of functionality, when subjected to appropriate controls to distinguish it from random conservation. *D. pseudoobscura *and *D. melanogaster *diverged some 25 to 55 million years ago, and have gone through sufficient divergence to scramble neutral sequences but yet not large enough to mask functional conservation [27]. Conservation of a whole ORF, from start to stop, appears to be a reasonably stringent filter because i) the number of surviving smORFs does not increase when the initial tBLASTn search is made less stringent; ii) surviving smORFs show syntenic conservation in very high numbers; and iii) the size distribution of the 4,561 conserved smORFs is significantly different from the starting pools and from random controls, whereas it does not change after applying further filters (Additional file 1). Some 4,500 smORFs might, therefore, constitute an upper estimate of the number of functional smORFs in *Drosophila*, which would be in line with the Hanada *et al*. [11] estimate of 3,241 smORFs in *Arabidopsis*.

It might be argued that syntenic conservation (that is, conservation in homologous regions of the genome) should be considered as the definitive criterion to distinguish real conservation (presumably due to functional significance) from simple similarity (presumably due to chance). However, the absence of synteny may be due to individual gene translocation. It is interesting that almost 50% of the 25 previously annotated and proteomics-corroborated smORFs are not conserved in syntenic positions in *D. pseudoobscura *(Table 3). Similarly, 178 of our smORFs are conserved with start and stop codons in *D. pseudoobscura*, have Ka/Ks < 0.1, and have multiple evidence of transcription, yet they do not appear to be conserved in syntenic positions (Figure 4). We note that both these 25 smORFs, plus another 6 for which we have separate evidence of function (E Magny and JP Couso, unpublished observations) belong to transcripts of less than 2 kb; perhaps small smORF-encoding transcripts are more mobile than their canonical counterparts. Taking into account these facts, we resolved not to consider synteny for our upper estimate of putative functional smORFs, but to include it amongst the criteria to generate our conservative estimate.

Following the conservative approach, we have taken the position that a functional smORF must also show evidence of both amino acid sequence conservation and transcription. We therefore studied first the pattern of sequence conservation in these conserved 3,144 syntenic smORFs, and observed that 1,075 smORFs show a Ka/Ks score < 0.1, which distinguish them from random conservation with a 10% FDR. Next, we assessed the correspondence between these 1,075 smORFs and transcribed regions in the *Drosophila *genome. Again using a conservative criterion, we observe that 401 of these smORFs overlap transcribed fragments on more than one occasion. These 401 smORFs are thus very strong candidates to be functional, as they present evidence of both transcription and amino acid conservation (see Additional files 3 and 4 for the actual sequences). Pending manual curation and functional studies, our preliminary clustering analysis suggests that those 401 smORFs may belong to 383 different transcripts, and therefore some 380 is our lower estimate for the number of smORF-containing genes in *Drosophila*. However, these may well be underestimates. Firstly, the experimental data of Manak *et al*. [36] and modENCODE that we have used here to ascertain transcription detects only transcripts expressed during embryogenesis. It leaves out transcripts only expressed during larval or adult phases of the life cycle, including organs transcriptionally active and rich in specific transcripts such as the brain and the gonads. Further, high-throughput detection of transcription during embryogenesis is not absolute but subject to inevitable experimental limitations. Thus, genes expressed in only a few cells of the embryo may not be represented (E Magny and JP Couso, unpublished observations). Secondly, the Ka/Ks test is of limited usefulness with short sequences, producing up to 9% false negatives [38] with a limit of Ka/Ks < 1. In order to obtain a 10% FDR, we have had to lower the Ka/Ks limit to 0.1, which is one order of magnitude lower than the usual < 1 used for longer protein sequences, and leaves out a further 1,868 smORFs (Table 2). It is also possible that the 314 smORFs with Ka/Ks > 1, may be genuine translated coding sequences undergoing adaptive evolution [54]. Finally, the average length and population size distribution of the 401 smORFs with evidence of transcription, synteny and Ka/Ks < 0.1 is not significantly different to that of the 4,561 conserved smORFs that have no evidence of synteny, transcription and Ka/Ks < 0.1 (Figure 2; Additional file 1), suggesting that some of the smORFs with no current evidence of transcription or protein sequence conservation may be transcribed at low levels, or at yet not fully explored stages of development and life history. Altogether, our conclusion from the Ka/Ks, transcription and synteny analysis is that our estimate of some 400 functional smORFs in *Drosophila *is solid but perhaps too conservative. A less conservative conclusion would be that up to 4,561 functional smORFs could exist. Between these numbers, other estimates are possible, depending on the actual filters and cutoff values considered (see Figure 4 for a breakdown of the results of applying each filter and of the different overlaps between these filters).

At any rate, a lower estimate of 400 smORFs represents some 3% of currently annotated protein-coding genes, and is in line with the 5% estimate in *Saccharomyces *[19], *Mus *[28] and a conservative *Arabidopsis *estimate. We therefore favor this conservative estimate for the number of functional smORFs in *Drosophila*.

### Obtaining further proof of smORF functionality

Full genome annotation, including manual curation, of the 401 smORFs conserved in *D. pseudoobscura*, overlapping transcripts, and with Ka/Ks < 0.1 is a necessary future step to build the gene models they belong to, but will not offer definitive proof of their functionality or translation, which can only come from experimental verification.

We provide a limited experimental functional validation of our computational search, pooling observations from previously published studies, and the results would seem to vindicate our computational estimates. The peptides for 25 smORFs in the annotated *D. melanogaster *genome with sizes ranging from 42 to 99 amino acids have been isolated by proteomics methods, and offer direct and unambiguous proof of their translation. In several cases these smORFs arise after splicing of multi-exon transcripts, and this limits the number of filters we can apply. However, it can be observed that, except for the evidence of multiple overlap with transcribed sequences, many of these smORFs pass any of our filters of tBLASTn E-value < 10^-3^, syntenic conservation, and Ka/Ks < 0.1 (Table 3). This test of our pipeline reveals it to be stringent, and validates our estimate of 401 functional smORFs in *Drosophila *as conservative.

It is possible that new proteomics approaches specifically designed to detect peptides of less than 100 amino acids [55,56] could offer definitive proof for the translation of these 401 smORFs. However, successful peptidomics approaches in insects, including *Drosophila*, have only been reported in a few cases and can only be attempted on an organ-by-organ and stage-by-stage basis [57-59]. Still, such proteomics and peptidomics studies routinely uncover high numbers of 'orphan' putative sequences that cannot be matched to annotated *Drosophila *proteins [40,56,60]. The problem is more serious with very short peptides, which offer fewer sequence signatures (digestion sites, and so no) for proteomic pipelines to obtain high-confidence sequences. These orphan sequences could be artifacts, or could be the products of non-annotated functional smORFs.

An alternative proof for the functionality of these smORFs in *Drosophila *could be obtained by genetic analyses. Single-smORF genetic studies can accurately characterize functional smORFs and are essential to build the gene models they belong to [22-24], but cannot be extended to a whole genome sweep because of restriction in methods, cost and time. Whereas functional genetic analysis is routine in *D. melanogaster*, standard genetic techniques are not suited to the analysis of hundreds of loci, which currently could only be attempted by a research consortium. Even in the yeast *S. cerevisiae*, with its much greater speed and ease of manipulation, Kastenmayer *et al*. [19] similarly pooled data from previous studies and their own mutational effort and were only able to detect a mutant growth phenotype in 22 out of 247 smORF mutants. Other types of phenotypes were not assessed, but it seems likely that more detailed studies are needed to increase the fraction of smORFs with known functions in this lower eukaryote (since the fraction of canonical genes showing a similar growth phenotype is only 25% [4]). Interestingly also, a staggering number of putative loci (16,383), defined by 18,873 mutant alleles, have still not been mapped to any *Drosophila *gene (FlyBase [21], FB2010_05 release 5 notes, accessed May 2010). Several of these mutants probably correspond to lost alleles of canonical genes. However, a significant fraction of them may in fact map to non-annotated genes and coding sequences, including smORFs.

Thus, it is likely that a specific research effort is needed to obtain experimental evidence for the translation and functionality of smORFs in *Drosophila*, probably the higher eukaryote model best suited for this, because of its ease of genetic manipulation and extensive genome annotation. This enterprise would be well worth the effort, judging from the results of the analysis of a single smORF-encoding gene, *tarsal-less*, which has prominent essential functions at several life stages and whose encoded peptides seem to work as cell signals [22-24], and the important functions of other smORF products such as antibacterial or sex peptides [60,61]. If the results of the bioinformatics search presented here are thus corroborated by further proteomic and functional studies in *Drosophila*, it would justify similar functional studies in vertebrates, where expense and time are even greater constraints. Since functional translated smORFs and smORF-encoding genes have now been shown to exist in *Saccharomyces *and *Drosophila*, they have all probability of existing in vertebrates as well. We do not necessarily expect that individual smORFs will be conserved in vertebrates (amongst other things, their small sizes make computational identification of homologues across distant species difficult if not impossible), but we expect a new class of genes encoding smORFs to be present; a new class, adding hundreds or even thousands of extra coding sequences to current genome annotations.

## Conclusions

Our results suggest that 400 functional smORFs remain to be characterized in *Drosophila*, and open the possibility that a further 4,000 functional smORFs may also exist. Results in other organisms echo our findings, and altogether suggest that short protein sequences constitute a significant, and largely uncharacterized, fraction of the gene products encoded by any genome. Characterization of these smORFs could have significant impacts on biology and medicine.

## Materials and methods

### Data used and smORF search

The intergenic DNA of *D. melanogaster *was downloaded from the FTP site of Flybase (release 5) [21]. We searched for ORFs that began with a start codon and ended with an in-frame stop codon within 30 to 300 bp on both strands of the *D. melanogaster *intergenic DNA. In doing so we only searched for unspliced ORFs. Due to their small size we called them smORFs (small ORFs) according to Basrai *et al*. [5]. We filtered from our analysis the smORFs that overlap with known coding DNA sequences, and transposon databases of *Drosophila*, as well as repetitive DNA.

### Control pool

As a control for our candidate smORF population we extracted from non-exonic *D. melanogaster *DNA a pool of 'reverse' smORFs running from stop to start. A 'reverse' smORF runs from a possible stop codon (TAA or TAG or TGA) to the next start codon (ATG). This extraction produced some 800,000 'reverse' smORFs and from them we randomly selected a sample of some 54,000 with the same size distribution as the 500 K smORF population. This control sample is thus composed of random DNA sequences comparable to (and not overlapping) our putative smORF population.

### Comparison with the genome of *D. pseudoobscura*

We translated the ORFs of *D. melanogaster *into amino acids using the universal genetic code. We searched for similarity between each translated smORF between *D. pseudoobscura *and *D. melanogaster *using standalone BLAST with the program tBLASTn. tBLASTn finds matches to an amino acid sequence within a nucleotide sequence by translating the nucleotide sequence in all three reading frames on both strands of the DNA duplex. It was run with default parameters, apart from an E-value threshold of 30. *D. melanogaster *and *D. pseudoobscura *are almost saturated at synonymous sites, indicating that there has been sufficient time for neutral sequences to almost completely diverge from one another.

We performed a FDR analysis to empirically estimate the proportion of smORF candidates that would have a given level of sequence similarity to the *D. pseudoobscura *genome purely by chance, and to choose tBLASTn E-value thresholds with an acceptable proportion of such false positives (that is, smORF candidates similarity above threshold by chance). We randomly sampled sequence segments from translated *D. melanogaster *exons, constraining these segments to have the same length distribution as the (translated) smORFs. We then used tBLASTn to find out the level of sequence similarity (represented by the E-value) between the *D. melanogaster *exon segments and *D. pseudoobscura*; for a given E-value threshold, we considered the proportion of exon segments that have E-value below the threshold 'true positives' (*TP*). We repeated the procedure using 'control smORFs' (sequences randomly sampled from the non-coding portion of the *D. melanogaster *genome, with the same length distribution as the smORFs), and considered the proportion of those with E-values below the threshold as 'false positives' (*FP*). We then computed the FDR for a given E-value threshold as *FP*/(*TP *+ *FP*).

For each smORF in *D. melanogaster*, which showed significant similarity (at the chosen FDR) to the genome of *D. pseudoobscura*, we investigated whether the sequence in *D. pseudoobscura *also contained an in-frame start and stop codon within 300 bp either side of the conserved amino acid sequence in the *D. pseudoobscura *genome. We extracted from *D. pseudoobscura *a further 300 bp immediately upstream of the tBLASTn hit, plus another 300 bp immediately downstream of it. The resulting 600+ nucleotide contig sequence was then re-aligned to the initial *D. melanogaster *smORF using ClustalW [62], and finally, start and stop codons in frame with the *D. melanogaster *smORF were sought in the re-aligned *D. pseudoobscura *sequence.

For each smORF we calculated non-synonymous substitutions per non synonymous site (Ka) and synonymous substitutions per synonymous site (Ks). We repeated the procedure described earlier to obtain an empirically estimated 10% FDR value for Ka/Ks, by computing Ka/Ks values for both annotated exon fragments and controls ('reverse smORFs').

### Database of transcribed regions

To investigate whether the putative smORFs were transcribed, we used the transcription data from Manak *et al*. [36]; in their experiment, RNA from fly embryos at different developmental stages was hybridized to genomic arrays with tiling probes of around 200 bp in length. Transcribed fragments located in non-annotated DNA were called 'transfrags' by the authors and we have maintained their nomenclature. To ascertain which of our smORFs are transcribed, we matched the coordinates of our putative smORFs with the transfrag coordinates kindly provided by J Manak. For RNASeq data, we used the data released by the modENCODE consortium for embryonic stages. Although data have been released for other stages, only the data for contigs of reads at embryonic stages indicate the DNA strand of origin, which is essential to ascertain a *bona fide *overlap with smORFs. We used the stringent criteria that to pass this filter, smORFs conserved in *D. pseudoobscura *must display multiple evidence of transcription (2,035 cases). 'Multiple evidence of transcription' means overlap with more than one RNA sequence in the right orientation - that is, overlap in more than one embryonic substage (1,928 cases) or in a single stage but overlapping two or more contig reads (23 cases), or finally, overlapping a single contig read but at least a transfrag as well (84 cases).

### Statistical analysis

Statistical analyses, including the Mann-Whitney and Kolmorogov-Smirnov tests, were performed using the SPSS package and R. Venn diagrams were generated using Venn diagram plotter (Pacific Northwest National Laboratory, US Department of Energy).

### Relaxed analysis of a sample of smORFs

Because the total number of smORFs isolated from the genome of *D. melanogaster *depends on the E-value for the tBLASTn search in *D. pseudoobscura *(Table 1), we performed a less stringent analysis using a subset of smORFs. Out of the 593,568 smORFs, we randomly sampled 10,000 smORFs from each chromosomal arm (2L, 2R, 3L, 3R and X) and 1,000 smORFs from chromosome 4, as it contains an order of magnitude fewer smORFs and euchromatin than other chromosomal arms (Table 1). We filtered these smORFs for coding DNA sequences, transposons and repetitive DNA and then we searched for homology with the genome of *D. pseudoobscura *using tBLASTn run with default parameters with an E-value threshold set to 0.05.

## Abbreviations

Bp: base pairs; FDR: false discovery rate; ORF: open reading frame; smORF: small open reading frame; uORF: upstream open reading frame.

## Authors' contributions

EL, VP, EM and JPC designed the experiments, obtained data, and analyzed and interpreted data. AEW designed experiments and interpreted data. All authors were involved in drafting and reviewing the manuscript. All authors have read and approved the manuscript for publication.

## Supplementary Material

Additional file 1 **Statistical analyses for the comparisons of smORF pools according to their codon length**. Click here for file

Additional file 2 **Cumulative size distributions of short exonic sequences before and after tBLASTn**. Click here for file

Additional file 3 **Fasta file with the sequences of the 401 smORFs representing our conservative estimate**. Click here for file

Additional file 4 **Excel file with the sequences of 401 smORFs representing our conservative estimate**. Click here for file
