# Supplementary material for: Hundreds of putatively functional small open reading frames in Drosophila
Source: Genome Biol. 2011 Nov 25;12(11):R118. doi: 10.1186/gb-2011-12-11-r118 (PMC3334604; doi:10.1186/gb-2011-12-11-r118)
Supplement: Additional file 1 — Statistical analyses for the comparisons of smORF pools according to their codon length. [file gb-2011-12-11-r118-S1.DOC]

Additional file 1. Comparisons of smORF pools according to their codon length:

**Mann-Whitney Test**

**Comparison: ALL non-conserved (556,558 – 43,197 =** 513,361) vs. tBLASTn hits (43,197)

| **Test Statistics^a^** | |
| --- | --- |
|  | lengthInNucl |
| Mann-Whitney U | 5.246E9 |
| Wilcoxon W | 1.370E11 |
| Z | -182.231 |
| **Asymp. Sig. (2-tailed)** | **.000** |

**Comparison:**

**tBLASTn hits (43,197) vs. Conserved smORFs (4,561)**

Wilcoxon rank sum test with continuity correction
**W = 162357406, p-value < 2.2e-16**

**Comparison: Conserved smORFs in Dp (4,561) vs. Conserved smORFs with Ka/Ks < 0.1 and multiple evidence of transcription (401)**

Wilcoxon rank sum test with continuity correction
**W = 666372.5, p-value < 2.2e-16**

| COMPARISON OF LENGTH DISTRIBUTIONS | Kolmogorov-Smirnov GOF test | Wilcoxon rank sum test |
| --- | --- | --- |
| smORFs vs. controls |
| after TBLASTN E<0.001 filter | D = 0.0893, p-value < 1e-10 | W = 35696968, p-value < 1e-15 |
| after TBLASTN E<0.05 filter | D = 0.0427, p-value < 0.0003 | W = 77201128, p-value = 1e-06 |
| after Start/Stop conservation (E<0.001) filter | D = 0.4546, p-value < 1e-15 | W = 182856, p-value < 1e-15 |
| after Start/Stop conservation (E<0.05) filter | D = 0.408, p-value < 1e-15 | W = 29087, p-value < 1e-15 |
