# Supplementary material for: Hundreds of putatively functional small open reading frames in Drosophila
Source: Genome Biol. 2011 Nov 25;12(11):R118. doi: 10.1186/gb-2011-12-11-r118 (PMC3334604; doi:10.1186/gb-2011-12-11-r118)
Supplement: Additional file 2 — Cumulative size distributions of short exonic sequences before and after tBLASTn. [file gb-2011-12-11-r118-S2.DOC]

Additional File 2, Figure S1. Cumulative size distributions of short exonic sequences before and after tBLASTn: Note that the starting sample of exonic sequences (green circles) has been selected to have the same size distribution as our 556K population of candidate smORFs. Increasing the stringency of tBLASTn (that is, lowering the E-value cut-off) discriminates against short sequences.

lengths of exon segments, before and after tBLASTn filter

cumulative density


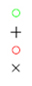


exon segments before filter

exon segments, E < 1e-5

exon segments, E < 1e-3

exon segments, E < 0.05

length (aa)

Additional file 2, Figure S1.
